# High use of commercial food products among infants and young children and promotions for these products in Cambodia

**DOI:** 10.1111/mcn.12270

**Published:** 2016-04-15

**Authors:** Alissa M. Pries, Sandra L. Huffman, Khin Mengkheang, Hou Kroeun, Mary Champeny, Margarette Roberts, Elizabeth Zehner

**Affiliations:** ^1^ Helen Keller International, Asia Pacific Regional Office Phnom Penh Cambodia; ^2^ Consultant to Helen Keller International; ^3^ Helen Keller International Phnom Penh Cambodia; ^4^ Helen Keller International Washington, D.C. USA

## Abstract

Despite national improvements in child survival, 40% of Cambodian children less than 5 years of age are stunted. Commercially produced complementary foods could be nutritionally beneficial for young children in Cambodia if fortified and of optimal nutrient composition. However, other nutrient‐poor commercially produced snack foods may be detrimental to young child feeding by displacing consumption of other nutritious foods. This study assessed consumption of commercial food products among infants and young children and their mothers' exposure to promotions for these products. A cross‐sectional survey was conducted among 294 mothers of children less than 24 months of age living in Phnom Penh. Of children 6‐23 months of age, 55.0% consumed a commercially produced snack food product on the prior day, and 80.6% had consumed one in the prior week. Only 12 (5.4%) children 6‐23 months of age had consumed a commercially produced complementary food. Almost all mothers (96.9%) had observed a promotion for a commercially produced snack food product, and 29.3% reported observation of a promotion for a commercial complementary food. Only one‐third (32.9%) of children 6‐23 months of age achieved a minimum acceptable diet. Findings indicate that there is a need to improve infant and young child feeding practices among children less than 24 months of age living in Phnom Penh. Nutritious options should be promoted, and consumption of unhealthy commercially produced snack food products should be discouraged.

**Key messages**

- Promotions for commercially produced snack food products are prevalent in Phnom Penh. As such products are likely detrimental to young child nutrition, there is a need to regulate advertisements. There is a need for research to illustrate the relationship of commercial snack food consumption with infant and young child nutrition.
- Consumption of commercially produced snack food products is common among children 6–23 months of age in Phnom Penh. Overconsumption of such foods can contribute to malnutrition, as well as adult obesity and non‐communicable disease. Nutrition interventions should encourage replacement of unhealthy snacks with more nutritious, affordable foods for young children.

## Introduction

While breastmilk is sufficient for optimal infant growth until the age of 6 months, after this time complementary foods need to be introduced in a safe and adequate manner, with continued breastfeeding. This period commonly corresponds to growth faltering in young children and is an important focus area for preventing future childhood malnutrition (Shrimpton *et al*. 2001). While Cambodia has focused efforts on public health interventions to improve child health, childhood undernutrition is a major problem. The 2010 Cambodia Demographic and Health Survey (CDHS) found that the percentage of children stunted increased with age, from 10% among children less than 6 months of age, to 19% among children aged 9–11 months and to 47% between 18 and 23 months of age (National Institute of Statistics (NIS) [Cambodia], Directorate General for Health and ICF Macro 2011).

Suboptimal complementary feeding practices in Cambodia remain a concern. In 2010, only 24% of Cambodian children aged 6–23 months met the minimum standard with respect to three infant and young child feeding indicators (feeding frequency, minimum diversity and consumption of breastmilk or other types of milk), with dietary diversity showing the most inadequacy in young children's diets (National Institute of Statistics (NIS) [Cambodia], Directorate General for Health and ICF Macro 2011). Only 37% of all children aged 6–23 months had been fed foods from the minimum number of food groups for their age. Adequate feeding frequency of infants aged 6–11 months was 62%, whereas dietary diversity (a minimum of four or more food groups) for infants aged 6–11 months was only 24% (Marriott *et al*. 2011).

Prior research also indicates that consumption of other commercially produced food products, often with poor nutritional quality, is prevalent among young children in Cambodia. One study among stunted toddlers living in Phnom Penh found that diluted sweetened condensed milk was frequently given as a source of milk for non‐breastfed children aged 12–42 months, along with other sugary drinks (Anderson *et al*. 2008). Anderson *et al*. also found that snack foods, such as crisps, biscuits and sponge cake, were given to infants and young children, with the purchase and consumption of these snacks often not supervised by an adult (Anderson *et al*. 2008). Another study in Kep province found that 87% of babies under 6 months of age who were not exclusively breastfed were fed solids, including snack cakes (Wren & Chambers 2011). Secondary analysis of the 2010 CDHS found that 44% of Cambodian children 6–23 months of age consumed sugary foods on the preceding day (Huffman *et al*. 2014). However, while prior research has assessed consumption of sugary snacks among young children, there is a need to measure consumption of the wider range of snack foods present in Cambodia, including sweetened beverages and savoury snacks.

Helen Keller International conducted a study to assess consumption of commercial food and beverage products among children less than 24 months of age in Phnom Penh, Cambodia, and to assess their mothers' exposure to commercial promotions for these products.

## Materials and methods

This study utilized a cross‐sectional survey with a multistage sampling procedure to obtain a representative sample of Phnom Penh mothers of children less than 24 months of age. Data were collected through structured interviews with Phnom Penh mothers. Sampling of participants and interviews were conducted at health facilities. Sampling within the health system served as a proxy for sampling mothers from the general population; health service utilization is high in Phnom Penh, with over 90% of Phnom Penh children accessing health facilities for basic vaccinations (National Institute of Statistics (NIS) [Cambodia], Directorate General for Health and ICF Macro 2011). As the age distribution of children present at health facilities was anticipated to be skewed to younger infants, sampling of mothers stratified children across age categories to allow for an equal age distribution that would be representative of the general Phnom Penh population. Mothers were asked to recall feeding practices of their youngest child and exposure to promotions of selected commercially produced foods since the birth of this child. Data were gathered from a period of November 2013 to February 2014.

Because variables of interest included breastfeeding practices, the study was limited to mothers and did not include other caregivers of children. Additionally, mothers with any of the following characteristics that could impede breastfeeding were excluded: mothers of children born with congenital diseases or who were in the neonatal intensive care unit; mothers who experienced severe delivery complications during the birth of their youngest child; and mothers whose youngest child was a twin or from a multiple birth. As prior studies have indicated commercial products to be more widely consumed in urban areas (Huffman *et al*. 2014), the study population included in this survey was limited to mothers currently living in and utilizing health facilities within Phnom Penh.

### Sample size

The sample size for this study was calculated to detect an anticipated prevalence of 10% of children's consumption of commercially produced food on the preceding day and 10% prevalence of mothers' exposure to promotions, with a measurement error of ±5%. Using a standard of error of 0.0255 and assuming a design effect of 2 to account for the cluster design, a sample size of 280 mothers of children less than 24 months of age was determined. A total of 498 mothers utilizing child health services at a facility were approached for interview. Ninety‐one (18.3%) of these mothers refused participation, and 113 (22.7%) mothers were excluded; 96 (19.3%) mothers lived outside of Phnom Penh, 11 (2.2%) mothers reported severe complications during delivery, 7 (1.4%) infants had been in the neonatal intensive care unit after delivery and 1 (0.2%) child was from a multiple birth. The majority of refusals by mothers were due to the mothers not having time and needing to leave the health facility after their child received services. The final sample included 294 mothers.

### Sampling procedure and data collection

**Figure 1:**
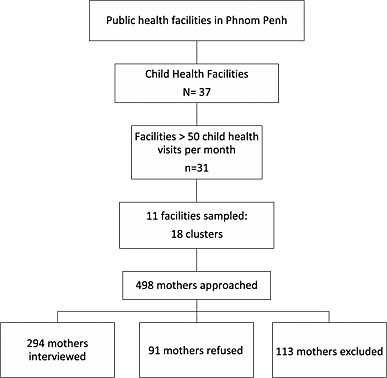
Sampling profile for mothers and facilities.

Lists of all public health facilities offering child health services were obtained from the Cambodian Ministry of Health. In addition, utilization rates for these facilities over the preceding year were also obtained, which included total number child health visits (outpatient department and immunization). Because of logistics and limited time for data collection, facilities with less than 50 child health visits per month were excluded from the sampling frame. The final sampling frame represented 97.3% of all child health visits in Phnom Penh public health facilities. All health facilities were then sampled by allocating clusters using probability proportional to size. Clusters of 16 mothers each were assigned across facilities in the sampling frame; the total of 16 mothers per cluster was chosen to allow for even distribution of child ages across four age categories (per cluster, four children were sampled in the following age ranges: 0–5.9, 6–11.9, 12–17.9 and 18–23.9 months). Eighteen clusters were sampled in the sampling frame of public facilities. Eleven public facilities were sampled; Fig. 1 details the sampling of facilities and mothers.

Staff at sampled facilities were alerted about data collection approximately 1 week prior to survey. Women with children at clinics offering child health services, either in the immunization or in the outpatient department, at a sampled health facility were approached for interview by survey supervisors. Survey supervisors screened every woman with a child who passed through the entrance/exit point of the child health clinic area. Women were first screened to assess (1) if they were the mother of the child with them, (2) if this child was under 24 months of age and (3) if they lived in Phnom Penh. If the required number of children in an age category had already been attained, mothers of children in this age category would not be interviewed.

Approval for this study was obtained from the Cambodia National Ethics Committee for Health Research prior to data collection. 1Approval was obtained on 28 August 2013 (Registration No. 0155/2013). Informed consent was obtained from all participants prior to the conducting of any interview.

### Questionnaire design

The questionnaires gathered data on mothers' characteristics, including age, marital status, educational attainment, household assets, work status and breastfeeding practices. Data collected on the youngest child included dietary intake on the day prior to interview. Data to assess infant and young child feeding practices were gathered in accordance with the WHO guidelines on infant and young child feeding practices; minimum dietary diversity was defined as a child consuming at least four of seven food categories in the previous day, minimum meal frequency was defined as a child consuming food the minimum number of times in the previous day, depending on their age and breastfeeding status, and minimum acceptable diet was defined as the combination of these two indicators (World Health Organization 2008). Additionally, data were gathered on the weekly frequency of consumption, reasons for feeding and expenditure for commercially produced snack food products hypothesized to be commonly fed to young children in Cambodia (based on a review of literature). Finally, mothers were also asked to report on promotional practices observed since after the birth of their youngest child.

The questionnaires were translated from English into Khmer, back translated into English to ensure accuracy and conducted in Khmer. Enumerators were locally recruited and attended a 5‐day training, which covered research objectives and detailed explanation of the questionnaire. Interviews were conducted using a mobile data collection system (Open Data Kit) using tablets in order to allow for immediate data entry, reduction in data errors and prompt analyses. Data were submitted online to a web‐based database (Formhub.org 2013) and were reviewed weekly to ensure data quality.

### Statistical analyses

Data were cleaned and analysed using spss version 21 (IBM, Armonk, NY, USA). Proportions were used to describe the samples. Differences in age categories and associations were assessed through bivariate comparison, using two‐sided Fisher's exact chi‐square test for proportions.

Commercially produced complementary foods were defined as any commercially produced food or beverage product, excluding breastmilk substitutes (infant formula, follow‐on formula or growing‐up milks), that contain a label indicating that the product is intended for children younger than 2 years of age. Types of commercially produced complementary foods include cereal/porridge, pureed food, snacks/finger food and tea/water/juice.

Commercially produced food products were defined as industrially manufactured foods intended for consumption among the general population, not only young children. These included instant noodles, commercial yogurt and commercial bread, as well as commercially produced snack food products. This latter category of products was defined as commercially produced and packaged foods typically eaten between meals. Savoury snack food products included fried chips, crisps or salted biscuits. Sugary snack food products included chocolates, sweets, candies pastries, cakes or sweet biscuits. Commercially produced sweetened beverages were defined to include soft drinks, branded juice drinks, condensed milk and chocolate/malted milks.

Commercial promotions were defined as any type of marketing technique intended to increase sales, including media or print advertising, provision of free samples or any other activity to encourage or induce the purchase of a product (IBFAN (International Baby Food Action Network) 2007). Exposure to such commercial promotion within the health system was measured by asking mothers if they had heard, seen or read any promotions since the birth of their youngest child, and if so, where they observed the promotion. Interpersonal promotions were also measured and were defined as recommendations/advice to use a commercial food product for infant and young child feeding.

## Results

### Demographics and socio‐economic characteristics

Nearly all mothers (98.6%, *n* = 290) were currently married. Over half of mothers (57.8%, *n* = 170) had attended any secondary school or higher. Approximately one‐fourth (24.1%, *n* = 71) of mothers reported currently working outside the home, and 83.0% (*n* = 244) were the main caregiver of the child. Only 15.0% (*n* = 44) of household did not have a television. Other demographic and socio‐economic characteristics of respondents are reported in Pries *et al*. (2016).

### Complementary feeding practices

Early introduction of semi‐solid foods was rare within this study sample; only four of 72 (5.6%) infants less than 6 months of age had been fed a soft/semi‐soft/solid food on the day prior to interview. Among 222 children 6–23 months of age, 14 children aged 6–23 months (6.3%) had not eaten food on the day prior to interview. Infant and young child feeding indicators relating to complementary feeding are shown in Table 1.

**Table 1:** Complementary feeding indicators among children 6–23 months of age, by age category and breastfeeding status

	Breastfed			Non‐breastfed		
	6–11 months old (*n* = 51)	12–17 months old (*n* = 49)	18–23 months old (*n* = 18)	6–11 months old (*n* = 22)	12–17 months old (*n* = 23)	18–23 months old (*n* = 59)
Minimum dietary diversity (%)	35.3	46.9	44.4	59.1	82.6	67.8
Minimum meal frequency (%)	78.4	75.5	88.9	18.2	43.5	32.2
Minimum acceptable diet (%)	33.3	38.8	38.9	13.6	43.5	28.8

Younger children were less likely to have received minimum dietary diversity in the previous day as compared with older children; only 42.5% of children 6–11 months of age were fed foods from the minimum recommended number of food groups, as compared with 58.3% of children 12–17 months of age and 62.3% of children 18–23 months of age (*P* = 0.037). Older children were less likely to consume the recommended minimum number of meals/snacks in the previous day compared with younger children; only 45.5% of children 18–23 months of age achieved minimum meal frequency, vs. 65.3% of children 12–17 months of age and 60.3% of children 6–11 months of age (*P* = 0.039). A minimum acceptable diet was achieved by one‐third (32.9%, *n* = 73) of children 6–23 months of age. There was no difference in minimum acceptable diet by age categories of children (*P* = 0.237). Twenty‐five per cent of children of mothers who had no formal education or attended only primary school were fed a minimum acceptable diet on the prior day, as compared with 38.8% of children of mothers who attended secondary school or higher (*P* = 0.031).

### Consumption of commercially produced food and beverage products

Utilization of commercially produced complementary foods, intended for consumption by children less than 24 months of age, was very rare among Phnom Penh mothers; only 5.4% (*n* = 12) of children 6–23 months of age had consumed a commercially produced complementary food on the day prior to interview. Eleven of these 12 children had consumed an infant cereal. Eight mothers of children 6–23 months of age (3.6%) reported that their child consumed a commercial vitamin on the day prior to interview, and no children 6–23 months of age had consumed a multiple micronutrient powder.

**Figure 2:**
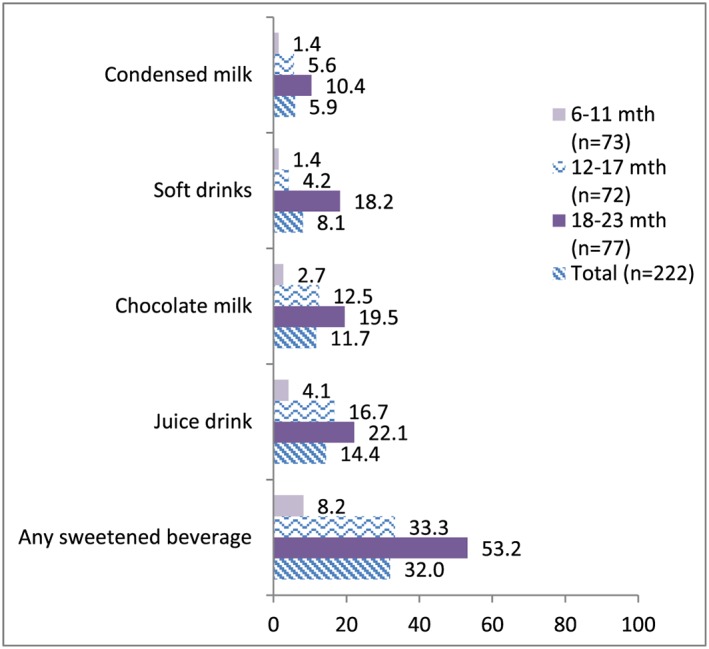
Percentage of children 6–23 months of age consuming commercially produced sweetened beverages for general consumption on the prior day (*n* = 222).

Consumption of commercially produced sweetened beverages, intended for consumption by the general population, was assessed among all mothers of children below 24 months of age. While no children below 6 months of age had consumed any of these commercial sweetened beverages, 32.0% of children 6–23 months of age had consumed one on the day prior to interview (Fig. 2). Across all categories of commercial sweetened beverages, consumption increased with age; 8.2% of children 6–11 months of age consumed any commercial sweetened beverage on the day prior to interview, compared with 33.3% of children 12–17 months of age and 53.2% of children 18–23 months of age (*P* < 0.001). Branded juice drinks and chocolate milks were the most commonly consumed sweetened beverages among children. Among children 18–23 months of age, 22.1% were fed branded juice drinks, 19.5% were fed chocolate milk, 18.2% were fed soft drinks and 10.4% were fed condensed milk.

**Figure 3:**
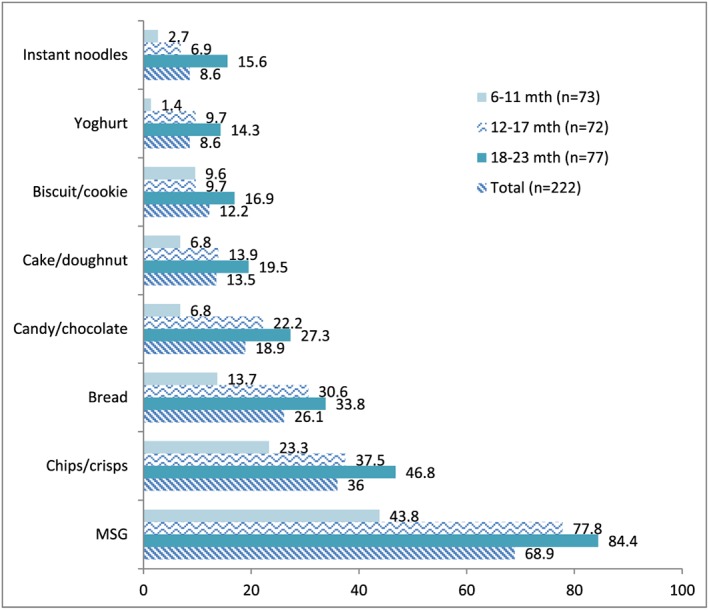
Percentage of children 6–23 months of age consuming commercially produced food products for general consumption on the prior day (*n* = 222).

**Figure 4:**
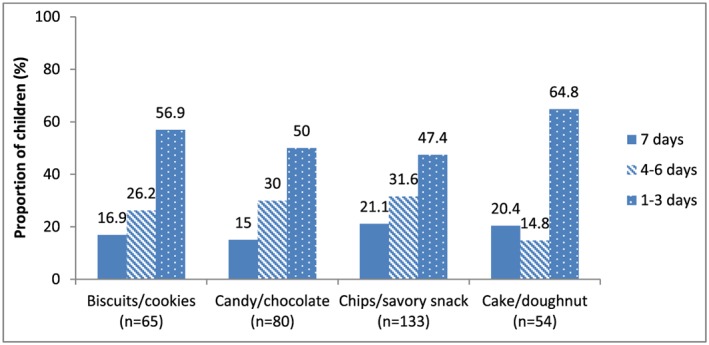
Frequency of commercially produced snack food product consumption among children 6–23 months of age in the week prior to interview.

Mothers were asked to report on their child's consumption of commercially produced food products in the day prior to interview among children 6–23 months of age (Fig. 3). About one‐fourth (26.1%) of children ate commercial bread, and less than 10% ate instant noodles or yogurt on the preceding day. Sixty‐nine per cent of children 6–23 months of age had consumed MSG in the prior day. Consumption of commercially produced snack food products was highly prevalent; overall, over half (55.0%, *n* = 122) of children 6–23 months of age had consumed a commercially produced snack food product in the day prior to interview, including biscuits/cookies, chips/crisps, cake/doughnuts or candy/chocolate. Eighty‐one per cent (*n* = 179) had consumed any commercially produced snack food product in the week prior to interview. Rates of consumption increased significantly with age; 38.4% of children 6–11 months of age, 58.3% of children 12–17 months of age and 67.5% of children 18–23 months of age consumed a commercially produced snack food product in the day prior to interview (*P* = 0.002). The majority (80.6%) of children 6–23 months of age had consumed a commercially produced snack food product in the week prior to interview. Consumption frequency of these products in the last week is shown in Fig. 4.

Consumption of savoury vs. sugary commercially produced snack food products was similar. Over one‐third (36.0%) had consumed a savoury snack product, such as chips or puffed rice crackers, and 36.5% had consumed a sugary snack product, such as cookies, candy or cake, in the day prior to interview. In the week prior to interview, savoury snacks were consumed daily by over one‐fifth of children 6–23 months of age (21.1%) who ate them in the last week, and 14.0% of children had consumed a sweet snack daily. Combining sweetened beverages with sweet snack food products, the per cent of children 6–23 months old who ate or drank any commercial sweet snack or beverage product on the preceding day was 50.0%.

**Figure 5:**
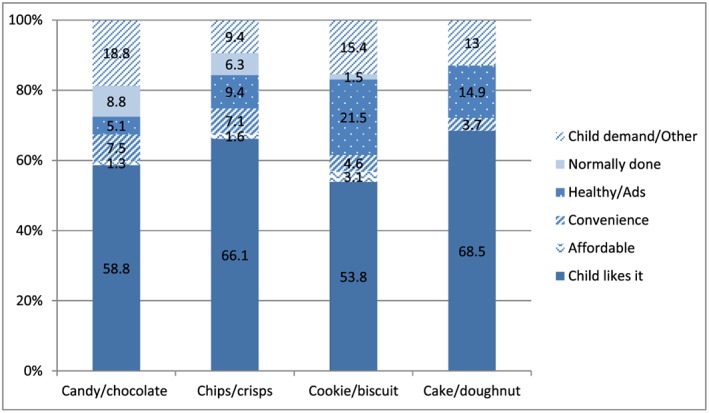
Reasons mothers of children 6–23 months of age fed commercial snack food products on the day prior to interview.

Mothers who had fed a commercially produced snack food product in the past week were asked to spontaneously report the main reason, as shown in Fig. 5. For all snack food products, the majority of mothers reported feeding the product to their youngest child because ‘the child likes it’, but many mothers reported that the child demanded it or ‘cried for it’. Twenty‐two percent of mothers who fed biscuits/cookies and 14.9% of mothers who fed cake/doughnuts reported doing so because they believed these products were healthy or saw advertisements that told them they were healthy for their child. When comparing mothers who did and did not work outside the home, there was no significant difference in the proportion that reporting doing so because of convenience (17.4% vs. 7.5%, *P* = 0.084). According to estimates by mothers, costs for commercially produced snack food products were minimal. Mothers who reported purchasing these products in the last week reported spending $0.20 per day on cookies/biscuits, $0.08 per day on candy/chocolate, $0.19 per day on chips/savoury snacks and $0.32 on cakes/doughnuts. There was no difference in the proportion of mothers that fed commercially produced snack food products based on socio‐economic characteristics of mothers including attainment of higher education (*P* = 0.411), refrigerator (*P* = 0.346) or car ownership (*P* = 0.606), employment outside the home (*P* = 1.000) or if the mother was the main caregiver of the child (*P* = 0.211).

### Promotions for commercially produced food products

Mothers of children less than 24 months of age were asked to recall if they had seen, heard or read a commercial promotion for various commercially produced foods since the birth of their youngest child, including commercially produced complementary food products and commercially produced snack food products. Twenty‐nine per cent (*n* = 86) of mothers reported observing a commercially produced complementary food promotion. Television was the most commonly reported source of promotions, reported by 84.9% of mothers who observed promotions. Infant cereals made up the majority of promotions reported for commercially produced complementary foods, and 17% (*n* = 15) of these reported promotions were for baby rusks. Five mothers (1.7%) reported promotions for ‘AD Milk’ brand, a sweetened liquid milk product that is not marketed specifically for children less than 2 years of age.

Almost all mothers (96.9%, *n* = 285) reported hearing, seeing or reading a promotion for a commercially produced snack food or beverage product since the birth of their youngest child. Reported promotions were highest for candy/chocolate (92.9%, *n* = 273) and savoury snacks (91.2%, *n* = 268). Over half (57.8%) of mothers of children 6–23 months of age who reported owning a television had fed their youngest child a snack food product on the day prior to interview, as compared with 36.7% of mothers who did not own a television (*P* = 0.047).

## Discussion

Results from this study indicate high consumption rates of commercially produced snack food products among children 6–23 months of age, and a high prevalence of commercial promotions for products, as reported by mothers living in Phnom Penh. Nearly all mothers of all children less than 24 months of age reported observing a promotion for a commercially produced snack food product. While promotions for commercially produced complementary foods were reported by mothers, utilization of commercially produced complementary foods was very low among mothers interviewed. Twenty‐nine per cent of mothers of children less than 24 months of age reported seeing, hearing or reading a promotion for a commercially produced complementary food product, with 84.9% of these promotions having been observed on television. Only 5.4% (*n* = 12) of mothers of children 6–23 months of age reported having fed a commercially produced complementary food, mainly an infant cereal, to their youngest child in the day prior to interview.

Among children 6–23 months of age, consumption of commercially produced snack food products was marked, including savoury snacks that are typically high in salt and sweet snacks that are typically high in sugar. The overall rate of snack food product consumption in this survey (55%) was much higher than that reported for urban areas (32%) in Cambodia in the 2010 CDHS (Huffman *et al*. 2014), which only measured consumption of sugary snack foods. The WHO recommends salt intake of less than 2 g per day because of the associations of high sodium consumption and increased risk of hypertension and cardiovascular disease (World Health Organization 2012). Research on sodium intake among Cambodians is limited. However, a recent study found mean sodium intake of 5.6 g across men and women in four provinces of Cambodia, including Phnom Penh (Ramsay 2014); this is 2.8 times higher than the WHO sodium intake recommendation. The high rates of salty snack food consumption among young children are of concern, as salt intake during early childhood has been shown to be correlated with high blood pressure in childhood (Strazzullo *et al*. 2012) and consumption of salt during infancy has also been shown to increase salt preference later on in life at 36–48 months of age (Stein *et al*. 2012). Similarly, research has shown that introduction to sugary foods early in life can influence preferences and consumption of sweet foods later in life (Avena *et al*. 2008; Ventura & Mennella 2011). Half of children 6–23 months of age in this study had consumed a sweet commercially produced snack food product or sweetened beverage on the preceding day. Beyond contributing to overweight/obesity during childhood, overconsumption of unhealthy foods early in life, such as commercially produced snack food products, can displace consumption of other important micronutrient‐rich foods and potentially contribute to undernutrition (Anderson *et al*. 2008). A number of studies have shown the association between malnutrition in early life and obesity risk later in life (Black *et al*. 2013; Huh *et al*. 2011).

There was no demonstrated association between mothers' observations of promotions for commercially produced snack food products and their children's consumption of these products; however, as almost all respondents had reported observing a promotion, it is believed that there were too few comparison cases to identify any association. Results also show a significant association between mothers' ownership of televisions and children's snack food product consumption. There is substantial research regarding television viewing and consumption of unhealthy foods among children, although most studies have been conducted in North America. A 2007 study by Powell *et al*. found that 97.8% of televised promotions for food products viewed by children 2–11 years of age were high in sodium, sugar or fat, indicating that almost all foods advertised were of poor nutritional quality (Powell *et al*. 2007). Another study analysing the content of television food advertisements aimed at children found that most messages depicted the products as being associated with fun and happiness, as well as a good taste (Folta *et al*. 2006). A review of food marketing by the Institute of Medicine found that televised marketing influences food preferences and purchase requests and short‐term consumption among children 2–11 years of age (Institute of Medicine (IOM) 2006). Most mothers in this study who fed commercially produced snack food products to their child reported doing so because the child liked the product or demanded the item, potentially indicating that even these young children have begun to develop food preferences, which could be related to marketing of these products.

While television promotions in this survey were observed by mothers, and potentially not their children, it is possible that advertisements targeting young children influenced mothers' perceptions of these products, including the belief that they are an appropriate food for their young child. When asked to report if they fed commercially produced complementary foods specifically for young children, 21.2% of mothers of children 6–23 months of age reported puffed rice crackers, which are manufactured for consumption by the general population and not solely children less than 24 months of age. Additionally, around one‐fifth of mothers who fed their children 6–23 months of age cookies/biscuits or cakes/doughnuts reported doing so because they believed these products were healthy for their child, with 7.7% of mothers who fed cookies/biscuits reporting that advertisements informed them that the product was healthy. Cambodia has a national policy to regulate the promotion of commercial infant and young child feeding products, specifically breastmilk substitutes and commercially produced complementary foods for children less than 24 months of age (Kingdom of Cambodia 2005), but this policy does not restrict promotion of other commercial food products that are commonly fed to young children, such as snack food products. To inform both policies and interventions, further research is needed to assess the impact of food advertisements on consumption patterns during the complementary feeding period and the general impact among populations in developing countries where exposure to promotions and utilization of commercially produced snack food products are increasing.

Indicators of quality of diet for children 6–23 months of age in this study were of concern; less than one‐third (32.9%, *n* = 73) of children 6–23 months of age in this study achieved a minimum acceptable diet, and only half (54.5%, *n* = 121) consumed the minimum recommendation for dietary diversity in the day prior to interview. The proportion of children achieving a minimal acceptable diet was particularly low among those 6–11 months of age who were not breastfed, at only 13.6%. Given the high prevalence of stunting among Cambodian children, ensuring consumption of nutritious, diverse foods of an appropriate quantity during the complementary feeding period is vital to improving child growth and development. If properly formulated for a young child and used appropriately during the complementary feeding period, fortified commercially produced complementary foods could play a positive role in the diets of young children in Cambodia. Among mothers included in this study, use of commercially produced complementary foods or multiple micronutrient powders was low. In 2011, Cambodia's Ministry of Health launched the Communication for Behavioural Impact (COMBI) Campaign to Promote Complementary Feeding in Cambodia, which promotes home preparation and feeding of enriched *borbor* (rice porridge) during the complementary feeding period. Twenty‐one per cent of children 6–23 months of age in this study had consumed home‐prepared *borbor* on the day prior to interview. This may indicate that among Phnom Penh mothers, home preparation and feeding of the traditional complementary food, *borbor*, is a more promoted and acceptable feeding practice than use of commercially produced complementary food products.

Addressing the high consumption of savoury and sugary snack food products and high rates of promotion should be a national priority. There is a need for interventions in Cambodia to encourage replacement of unhealthy snacks with more nutritious, affordable foods, in order to improve infant and young child nutritional status and to prevent increases in childhood overweight and obesity. Efforts to ensure adequate nutrition for infants and young children would serve to combat malnutrition during childhood, with the potential to positively impact adult overweight/obesity and associated non‐communicable diseases.

There are several limitations to this study to be considered. First, the working status of many mothers in Phnom Penh could influence findings from this study. The majority of refusals for participation in the survey were due to mothers who reported not having time for participation; it is possible that these were employed mothers who needed to return to work, and this study sample could have therefore been skewed to include a lower proportion of working mothers than that of the general Phnom Penh population. Utilization of commercial food products for infant and young child feeding could differ between mothers depending on work status; however, there was no significant difference in feeding of commercially produced infant cereal (*P* = 0.512) or snack food products (*P* = 1.000) between mothers who worked outside the home and those who did not. Additionally, while 24.1% of mothers in this study reported working outside the home, other caregivers were not interviewed regarding child consumption; the dietary intake data based on maternal recall of the day prior to the interview could be biased, as working mothers may not entirely know what the child had consumed over the previous day.

## Source of funding

The Bill & Melinda Gates Foundation funded the study.

## Conflicts of interest

The authors have no conflicts of interest to declare.

## Contributions

AP analysed the data and prepared the manuscript. SH and EZ conceptualized and designed the study, with input from HK. MC oversaw questionnaire development and technology for data collection. KM oversaw data collection. MR oversaw enumerator training. All authors reviewed and provided input on the final article.

## References

[mcn12270-bib-0001] Anderson V. , Cornwall J. , Jack S. & Gibson R. (2008) Intakes from non‐breastmilk foods for stunted toddlers living in poor urban village of Phnom Penh, Cambodia are inadequate. Maternal & Child Nutrition 4 (2), 146–159. 18336647 10.1111/j.1740-8709.2007.00120.x PMC6860648

[mcn12270-bib-0002] Avena N.M. , Rada P. & Hoebel B.G. (2008) Evidence for sugar addiction: behavioral and neurochemical effects of intermittent, excessive sugar intake. Neuroscience and Behavioral Reviews 32 (1), 20–39. 10.1016/j.neubiorev.2007.04.019 PMC2235907 17617461

[mcn12270-bib-0003] Black R. , Victora C. , Walker S. & Maternal and Child Nutrition Study Group (2013) Maternal and child undernutrition and overweight in low‐income and middle‐income countries. Lancet 382 (9890), 427–451. 23746772 10.1016/S0140-6736(13)60937-X

[mcn12270-bib-0004] Folta S.C. , Goldberg J.P. , Economos C. , Bell R. & Meltzer R. (2006) Food advertising targeted at school‐age children: a content analysis. Journal of Nutrition Education and Behavior 38 (4), 244–248. 16785094 10.1016/j.jneb.2006.04.146

[mcn12270-bib-0005] Formhub.org . (2013) Formhub. [online] Available at: http://formhub.org [Accessed 24 Apr. 2013].

[mcn12270-bib-0006] Huffman S. , Piwoz E. , Vosti S. & Dewey K. (2014) Babies, soft drinks and snacks: a concern in low‐and middle‐income countries? Maternal & Child Nutrition 10 (4), 562–574. 24847768 10.1111/mcn.12126 PMC4299489

[mcn12270-bib-0007] Huh S. , Rifas‐Shiman S. , Taveras E. , Oken E. & Gillman M. (2011) Timing of solid food introduction and risk of obesity in preschool‐aged children. Am Acad Pediatrics. 127 (3), e544–e551. 10.1542/peds.2010-0740 PMC3065143 21300681

[mcn12270-bib-0008] IBFAN (International Baby Food Action Network) (2007) Code Monitoring Kit. IBFAN Sdn Bhd: Penang.

[mcn12270-bib-0009] Institute of Medicine (IOM) (2006) National Academy of Sciences, Committee on Food Marketing and the Diets of Children and Youth In: Food Marketing to Children and Youth: Threat or Opportunity? (eds McGinnis J.M., Gootman J. & Kraak V.I.). Institute of Medicine of the National Academies: Washington, DC.

[mcn12270-bib-0010] Kingdom of Cambodia . (2005) Sub‐decree on the marketing of products for infant and young child feeding (No.133).

[mcn12270-bib-0011] Marriott B.P. , White A. , Hadden L. , Davies J.C. & Wallingford J.C. (2011) World Health Organization (WHO) infant and young child feeding indicators: associations with growth measures in 14 low‐income countries. Maternal and Child Nutrition 8 (3), 354–370. 22171937 10.1111/j.1740-8709.2011.00380.x PMC6860880

[mcn12270-bib-0012] National Institute of Statistics (NIS) [Cambodia], Directorate General for Health and ICF Macro (2011) Cambodia Demographic and Health Survey 2010. National Institute of Statistics, Directorate General for Health, and ICF Macro: Phnom Penh, Cambodia and Calverton, Maryland, USA.

[mcn12270-bib-0013] Powell L.M. , Szczypka G. , Chaloupka F.J. & Braunschweig C.L. (2007) Nutritional content of television food advertisements seen by children and adolescents in the United States. Pediatrics 120 (3), 576–583. 17766531 10.1542/peds.2006-3595

[mcn12270-bib-0014] Pries A.M. , Huffman S.L. , Mengkheang K. , Kroeun H. , Champeny M. , Roberts M. *et al.* (2016) Pervasive promotion of breast‐milk substitutes in Phnom Penh, Cambodia and high usage by mothers for infant and young child feeding. Maternal and Child Nutrition. (2016), 12 (Supp. 2), pp 38–51. 27061955 10.1111/mcn.12271 PMC5071766

[mcn12270-bib-0015] Ramsay L.C. (2014) A cross‐sectional evaluation of sodium consumption by people in Cambodia. M.Sc. Thesis. University of Guelph: Canada.

[mcn12270-bib-0016] Shrimpton R. , Victora C. , de Onis M. , Lima R. , Blossner M. & Clugston G. (2001) Worldwide timing of growth faltering: implications for nutritional interventions. Pediatrics 107 (5), 75–75. 10.1542/peds.107.5.e75 11331725

[mcn12270-bib-0017] Strazzullo P. , Campanozzi A. & Avallone S. (2012) Does salt intake in the first two years of life affect the development of cardiovascular disorders in adulthood? Nutrition, Metabolism, and Cardiovascular Diseases 22 (10), 787–792. 10.1016/j.numecd.2012.04.003 22749679

[mcn12270-bib-0018] Stein L.J. , Cowart B.J. & Beauchamp G.K. (2012) The development of salty taste acceptance is related to dietary experience in human infants: a prospective study. American Journal of Clinical Nutrition 95 (1), 123–129. 22189260 10.3945/ajcn.111.014282 PMC3238456

[mcn12270-bib-0019] Ventura A.K. & Mennella J.A. (2011) Innate and learned preferences for sweet taste during childhood. Current Opinion in Clinical Nutrition and Metabolic Care 14 (4), 379–384. 21508837 10.1097/MCO.0b013e328346df65 PMC12974590

[mcn12270-bib-0020] World Health Organization (2008) Indicators for Assessing Infant and Young Child Feeding Practices, Part 1‐definitions: Conclusions of a Consensus Meeting Held 6–8 November 2007 in Washington D.C., USA. Geneva WHO.

[mcn12270-bib-0021] World Health Organization (2012) Guideline: Sodium Intake for Adults and Children. World Health Organization: Geneva, Switzerland. 23658998

[mcn12270-bib-0022] Wren H. & Chambers L. (2011) Breastfeeding in Cambodia: mother knowledge, attitudes and practices. World Health Population 13 (1), 17–29. 22543417 10.12927/whp.2011.22498

